# Supplementary figures and images for: The development of resistance to an inhibitor of a cellular protein reveals a critical interaction between the enterovirus protein 2C and a small GTPase Arf1
Source: PLoS Pathog. 2023 Sep 18;19(9):e1011673. doi: 10.1371/journal.ppat.1011673 (PMC10538752; doi:10.1371/journal.ppat.1011673)

|              | 2C             |                |                |                |                 |
|--------------|----------------|----------------|----------------|----------------|-----------------|
|              | C4200T<br>S26L | A4318C<br>Q65H | C4339A<br>H72Q | T4410C<br>V96A | A4542G<br>N140S |
| 59/2C        | *              | *              | *              | *              | *               |
| 59/2C/-S26L  |                | *              | *              | *              | *               |
| 59/2C/-Q65H  | *              |                | *              | *              | *               |
| 59/2C/-H72Q  | *              | *              |                | *              | *               |
| 59/2C/-V96A  | *              | *              | *              |                | *               |
| 59/2C/-N140S | *              | *              | *              | *              |                 |

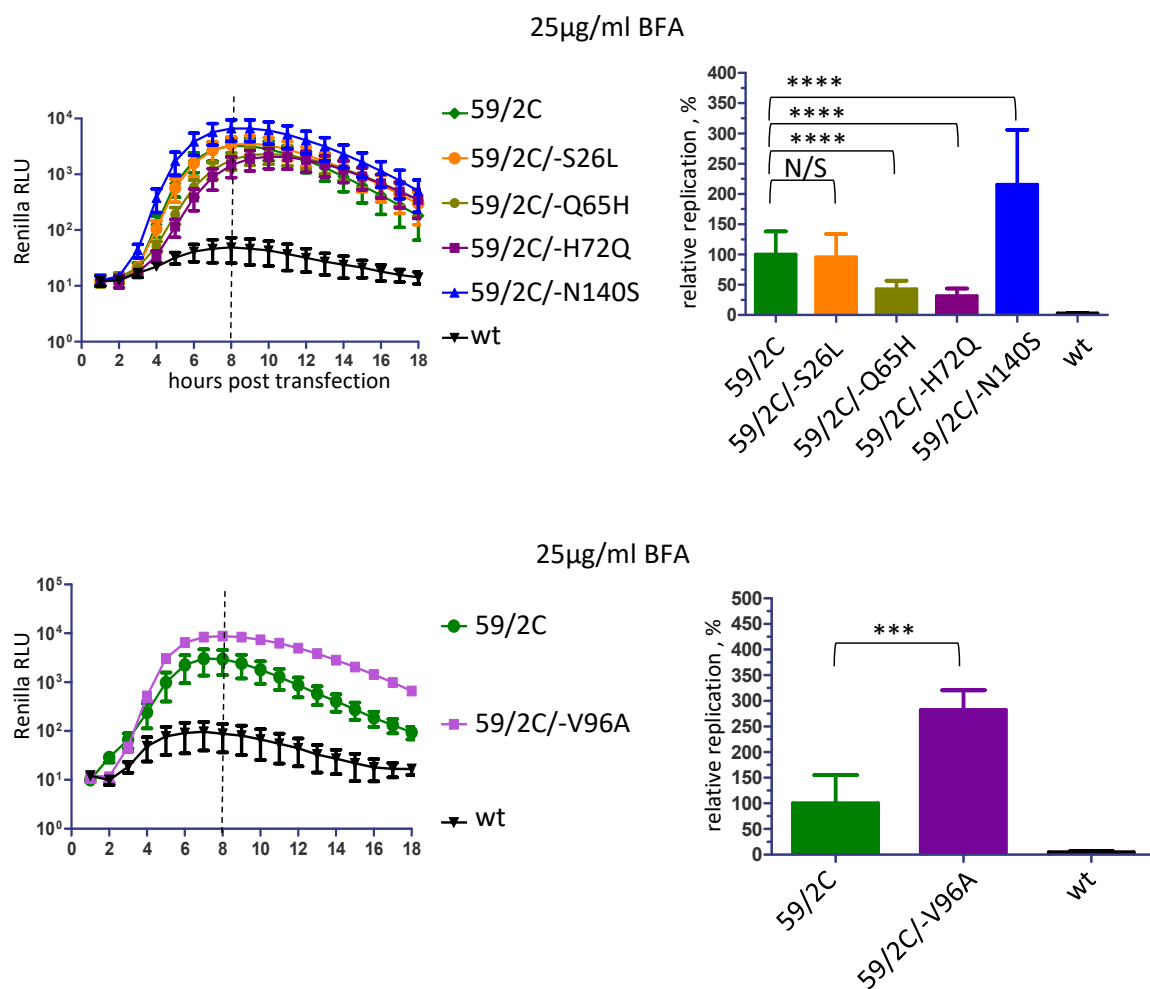

Figure S1

Supplement: S1 Fig — Relative replication is calculated for the signal up to 8 h post-transfection (dashed line) and is normalized to the replication of the replicon with the full complement of resistant mutations in 2C (59/2C). (PDF) [file ppat.1011673.s001.pdf]

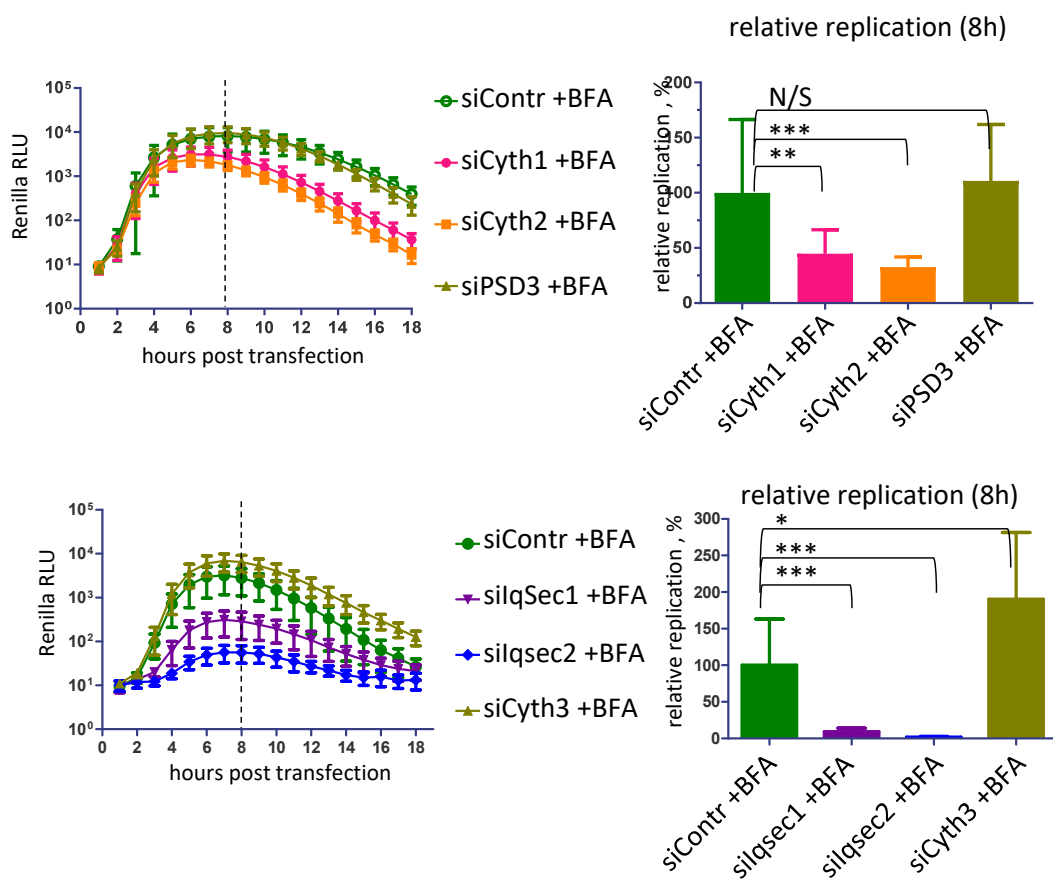

Figure S2

Supplement: S2 Fig — Relative replication is calculated for the signal up to 8 h post-transfection (dashed line) and is normalized to the replication of in cells treated with control siRNA. (PDF) [file ppat.1011673.s002.pdf]
